# Study of different acute SIT protocols’ impact on 200m sprinters’ anaerobic performance

**DOI:** 10.3389/fphys.2025.1672978

**Published:** 2025-09-11

**Authors:** Ying Liu, Qi Liu, Zhigang Gong, Junqian Yan, Juntao Yan

**Affiliations:** ^1^ College of Education, Beijing Sport University, Beijing, China; ^2^ Faculty of Physical Education, Jiangxi Normal University, Nanchang, China; ^3^ Tsinghua University High School Daxing School, Beijing, China

**Keywords:** sit, sprint interval training, anaerobic capacity, sprint running, surface electromyography

## Abstract

**Introduction:**

Sprint interval training (SIT), characterized by its “all-out” maximal-intensity efforts, elicits substantial energy output in a short duration, demonstrating superior anaerobic performance. This study compared the acute effects of different SIT protocols to identify the optimal training combination for enhancing anaerobic capacity in 200-m sprinters.

**Methods:**

Twelve male 200-m sprinters performed SIT sessions in a 2 (sprint duration: 20s, 30s) × 3 (load: 7.5%, 9%, 10% body mass (BM)) × 4 (repetitions) design with 4-min inter-set rest, assessed via anaerobic power, electromyography (EMG), and blood lactate measures.

**Results:**

(1) Duration and load significantly affected peak power (PP), mean power (MP), and fatigue index (FI) (P < 0.05); (2) Sprint duration significantly influenced lower-limb integrated EMG (IEMG) and median frequency (MPF) (P < 0.05), with no notable interaction effects; (3) Both duration and load significantly modulated muscle activation (RMS%) in the rectus femoris, biceps femoris, and vastus lateralis (P < 0.05), but not in the gastrocnemius; (4) Duration significantly increased maximal blood lactate, accumulated lactate, and rating of perceived exertion (RPE) (P < 0.05), whereas load had no independent effect; (5) The interaction between duration and load exhibited highly significant effects on anaerobic performance (P < 0.01).

**Conclusion:**

A high-intensity SIT protocol comprising 4 × 20s all-out sprints at 10%BM load with 4-min rest intervals optimally enhances 200-m sprinters’ anaerobic capacity through multidimensional physiological stimuli, providing an effective training strategy for performance optimization.

## Highlight

• A sprint interval training (SIT) protocol of 4 × 20-s maximal sprints at 10% body mass (BM) load with 4-min recovery effectively demonstrates anaerobic capacity in 200-m sprinters.
• The protocol significantly stimulates both the ATP-PCr (phosphagen) system and glycolytic pathways, demonstrating superior peak power output and speed endurance performance.
• This SIT protocol boosts 200 m sprint performance by activating key muscles and increasing lactate production.

## 1 Introduction

Sprint interval training (SIT) refers to an “all-out” sprint training protocol with durations ≤45 s, interspersed with active recovery periods between sets (Buchheit and Laursen, 2013). Its most distinctive feature is that the work intervals are typically performed at maximal (all-out) intensity, placing SIT at the highest end of the intensity spectrum within high-intensity interval training methodologies (Gibala et al., 2012). Existing evidence suggests that certain SIT protocols can improve body composition, aerobic capacity, and anaerobic performance across diverse populations; however, not all SIT interventions yield these benefits, likely due to variations in work interval design and loading parameters, leading to divergent outcomes. Currently, the most classic SIT protocol involves 8–30 s of maximal-effort cycling on a Wingate ergometer with an external load of 5%–10% body mass (BM) (Grgic, 2022), interspersed with 3–5 min of low-intensity active or passive recovery, performed in 4–6 sets per session, 3 sessions per week, for 4–6 weeks (Li, 2022). Nevertheless, the differences in internal load (physiological stress), external load (mechanical work), and energy expenditure characteristics between this traditional SIT protocol and alternative combinatorial regimens require further indepth investigation (Hazell et al., 2010; Gillen et al., 2014; Gillen et al., 2016; Cantrell et al., 2014).

The 200-m sprint is a physically demanding event primarily fueled by anaerobic metabolism, placing high demands on both the phosphagen and glycolytic energy systems, requiring athletes to move at maximum speed and cross the finish line at their fastest pace. Scholar Yuan (2006) pointed out that in men’s 200-m races, the ATP-CP system contributes approximately 23%–43% of energy supply, the glycolytic system accounts for 60%–63%, while aerobic metabolism constitutes only about 10%. Given these findings, enhancing anaerobic metabolic pathways is crucial for improving 200-m performance and holds practical significance. However, the classic 30-s sprint interval duration is slightly longer than the actual 200-m race time, and there is limited research on SIT specifically for 200-m sprinters. Currently, it remains unclear which SIT protocols would yield optimal benefits when applied to 200-m event preparation.

Beneke et al. (2002) demonstrated that the total energy expenditure of Wingate-based SIT is 128.1 kJ/30s, primarily fueled by the glycolytic system. Therefore, Wingate-based SIT training is an exercise predominantly targeting the development of glycolytic energy contribution, which benefits speed oriented athletes by enhancing anaerobic glycolytic capacity and neuromuscular function, while also contributing to neural and muscular adaptations induced by strength training to a certain extent. Research indicates that 2 weeks of SIT with 4–7 × 30-s all-out sprints can enhance endurance performance and improve resting skeletal muscle metabolism (Burgomaster et al., 2005). Meanwhile, an SIT protocol of 6 × 30-s sprints (with 4.5-min rest intervals) without fixed load effectively increases maximal oxygen uptake and submaximal exercise thresholds (Inglis et al., 2024). Biomechanically, the transfer effect between Wingate test and running performance primarily manifests in power output patterns of the hip, knee, and ankle joints. Both activities demonstrate similar triple extension dynamics during maximal power output (Korff et al., 2007). Studies reveal comparable hip angular velocities at peak power between Wingate cycling and sprint acceleration, with highly consistent EMG activation timing in vastus lateralis and biceps femoris muscles, establishing a neuromuscular basis for cross-sport anaerobic capacity transfer (Bezodis et al., 2014). Additionally, cycling-based sprint interval training protocols provide sufficient training stimuli to induce transfer effects on running performance (Kavaliauskas et al., 2015). These findings collectively suggest that non-specific training can help improve running performance in already trained runners.

Previous studies have indicated that current research on the effects of acute experimental variables and their interactions remains in the early stages (Benítez-Flores et al., 2018; Takeda et al., 2024; Lloria-Varella et al., 2023). Simultaneously, there is limited research concerning sprint interval training’s impact on 200 m sprinters, and the influence of related variables on these athletes’ anaerobic capacity has yet to be conclusively determined, which restricts the applicability of research findings to this specific population. This study compares the effects of different sprint interval training protocols under acute testing conditions to identify the optimal training combination for enhancing anaerobic capacity in 200 m sprinters. By analyzing the training benefits of various SIT modalities, we aim to optimize training parameters and provide scientific training guidelines for athletes, thereby improving anaerobic metabolic capacity and athletic performance. It was hypothesized that SIT with 20-s sprints and a 10%BM load would lead to superior anaerobic performance.

## 2 Materials and methods

### 2.1 Subjects

This study takes the influence of different combinations of sprint intervals on the anaerobic capacity of 200 m sprinters as the research object. Using G*Power 3.1 for sample size calculation, with reference to previous studies, the effect size was set at 0.4, statistical power at 0.8, and significance level at 0.05 (Teng et al., 2017) resulting in a calculated required sample size of 7 subjects. Considering potential subject attrition and data validity, the study ultimately recruited 12 national level athletes (McKay et al., 2022). Inclusion criteria comprised: male subjects aged 18–24 years; in good health without known cardiovascular diseases or other chronic conditions, and free from sports injuries for the past 3 months; being competitive 200-m sprinters; voluntarily participating in the study with commitment to complete all training sessions and tests. Exclusion criteria included: participation in other high-intensity training programs; presence of chronic diseases or acute sports injuries; and current medication use.

Prior to the study initiation, all participants were informed of the potential benefits and risks of the research, learned and familiarized themselves with the bicycle ergometer and the RPE scale, and signed the informed consent form after a detailed explanation of the experimental procedures. The experimental period runs from March 1 to 15, 2025, during which each subject undergoes one SIT test at a fixed time every other day, completing a total of six tests with different combinations (Lloria-Varella et al., 2023), (Mendez-Villanueva et al., 2007). All the arrangements of the plans were randomly grouped according to SPSS27.0, and the subjects were not aware of the duration and load of the sprint. Each person will take the test six times in total, with a 1-day interval between each test. All testing sessions were carried out in an air-conditioned laboratory where temperature was maintained at 20 °C and relative humidity controlled between 60% and 70% to minimize environmental variability. This study was approved by the Ethics Committee of Sports Science Experiments at Beijing Sport University (Approval No. 2025137H) and strictly adhered to the principles outlined in the Declaration of Helsinki. The baseline characteristics of subjects are presented in Table 1.

**TABLE 1:** Basic information of the Subjects (n = 12).

Age (y)	Height (cm)	Body mass (kg)	Years of training (y)
19.42 ± 1.98	178.75 ± 4.22	72.25 ± 5.36	5.08 ± 1.0

### 2.2 Measurements

This study employed a two-factor (2 durations × 3 loads) experimental design, in which subjects performed all-out cycling sprints on a Wingate ergometer (Cyclus2, Germany) with two sprint durations (20s and 30s) and three load factors (7.5%BM, 9%BM, and 10%BM). Each session consisted of 4 sets with 4-min recovery intervals between sprints. Capillary blood samples were collected from fingertips to measure blood lactic acid (BLA) concentration before warm-up. Following the final sprint, BLA levels were measured immediately post-exercise, at 3 min, and at 5 min using a portable lactate analyzer (EKF C-line Clinic, Germany), while perceived exertion was assessed using the Borg 6–20 scale. Surface electromyography (Delsys Tringo 16-channel wireless EMG system, United States) was used to continuously monitor muscle activity of the rectus femoris (RF), vastus lateralis (VL), biceps femoris (BF), and gastrocnemius medialis (GM) throughout all sessions. Each training session began with a 15-min warm-up, including dynamic stretching, movement integration, and neural activation, the results demonstrated that dynamic stretching significantly improves power output and concluded with 10 min of cool-down stretching exercises (Franco et al., 2012) (Unick et al., 2005).

#### 2.2.1 Surface electromyography (sEMG) testing

##### 2.2.1.1 Electrode placement

Electrode placement was determined through comprehensive analysis of joint kinematics, muscle functional characteristics, lower limb cycling biomechanics, and literature review to identify the principal muscle groups involved in pedaling. RF serves as the primary muscle responsible for both hip flexion and extension, while the VL and BF constitute the principal muscle groups governing knee flexion and extension, with the GM acting as the dominant muscle for ankle flexion and extension movements. sEMG analysis of quadriceps/hamstring activation timing identified the right limb as the dominant lower extremity. Following theoretical screening, four right lower limb muscles (RF, VL, BF, and GM) were targeted for electrode placement. The Ag-Cl electrode pair was positioned with 15–20 mm inter-electrode spacing, acquiring signals at 1,000 Hz sampling frequency with CMRR exceeding 80 dB. Standardized skin preparation included disinfection with 75% ethanol at predetermined muscle sites, followed by shaving to remove cutaneous oils and stratum corneum to minimize impedance. The EMG electrodes were then placed parallel to the muscle fiber direction on the muscle belly. To minimize motion artifact interference, we secured the electrodes with elastic bandages for stabilization (Stegeman and Hermens, 2007) (McManus et al., 2021). The pasting positions are shown in Table 2 (Jianhua and Wang, 2015).

**TABLE 2:** Placement site for EMG electrodes.

Muscle name	Electrode position
rectus femoris	Midpoint between ASIS and patella’s upper border
vastus lateralis	Distal two-thirds of ASIS-to-lateral patella line
biceps femoris	Midpoint of ischial tuberosity-lateral tibial condyle line
gastrocnemius medialis	Over the maximal muscle belly prominence

Note: ASIS, anterior superior iliac spine.

##### 2.2.1.2 Data processing

The raw sEMG signals were processed and analyzed using the built-in EMG signal processing software of the Delsys Tringo sEMG testing system. The EMG Server software sequentially processed the raw EMG data through filtering, rectification, and smoothing procedures. Specifically, the selected raw EMG segments were subjected to band-pass filtering (30Hz–480 Hz), followed by signal rectification to obtain absolute values. To account for inter-individual variability, the processed data were normalized using the maximal normalization method, yielding three key parameters: integrated EMG (iEMG), root mean square% (RMS%), and mean power frequency (MPF) (Dubo et al., 1976). Compared to static MVC (Maximal Voluntary Contraction), using the maximum values obtained from dynamic testing (Wingate test) better reflects the physiological characteristics of actual movement (Wakeling et al., 2001). In this normalization approach, the maximum values obtained from each subject’s Wingate test for individual muscles served as the reference values, with the specific formulas applied as follows (Fu, 2011; Guopeng et al., 2020):

$$\text{Normalized iEMG}=\text{iEMG }\left(\text{measured value}\right)\;/\text{ Maximum iEMG}$$

RMS%=RMS value÷Maximum RMS of individual muscle×100×100%

#### 2.2.2 Wingate testing

Prior to testing, experimenters instructed participants regarding the testing protocol, movement requirements, and safety precautions during cycling. Participants performed 2–4 min of general warm-up exercises followed by specific warm-up on the cycle ergometer, while research staff monitored surface EMG signal quality. The subjects cycled for 5 min, during which they performed 3 rapid sprints lasting 4–8 s at a load of 7.5% BM (Bar-Or, 1987) (Mastalerz et al., 2024). Upon hearing the “start” command from the experimenter, the formal test began. Throughout testing, participants received continuous verbal encouragement to maintain maximal effort while remaining seated. The procedure yielded three key performance metrics: peak power (PP), mean power (MP), and fatigue index (FI). FI was calculated using the following specific formulas (Dotan, 2006):

$$\text{FI}\%=\left(\text{Peak Power }–\text{ Minimum Power}\right)\;/\text{ Peak Power}\times 100\%$$

#### 2.2.3 Blood lactate sampling

Capillary blood samples (10 μL) were collected from fingertips at rest and immediately post-SIT, as well as at 3-min and 5-min intervals post-exercise (Polat, 2016) (Gültekin et al., 2006). All samples were analyzed within 2 h of collection using a benchtop lactate analyzer. The maximum blood lactate concentration was determined as the highest value among measurements taken immediately, at 3 min, and at 5 min post-SIT. The accumulated blood lactate was calculated using the formula: Accumulated blood lactate = Maximum blood lactate - Pre-SIT lactate (measured after warm-up), with all values expressed in mmol/L.

#### 2.2.4 Rating of perceived exertion (RPE) testing

The Borg 6–20 scale was employed to monitor internal training load, with participants’ RPE collected pre-exercise, immediately after each exercise bout, and at 2-min and 5-min intervals following each training set. The scale anchors were defined as follows: 6 (“no exertion at all”), 12 (“somewhat hard”), 16 (“very hard”), and 20 (“maximal exhaustion”) (Borg, 1982).

### 2.3 Statistical analyses

All data in this study were recorded using Microsoft Office Excel and analyzed with SPSS 27.0 software, while figures were generated using GraphPad Prism 10.1. Descriptive statistics were performed for all experimental indicators, with results expressed as mean ± standard deviation (Mean ± SD). All statistical tests were two-tailed, with significance levels set at P < 0.05 and high significance at P < 0.01. Data normality was first assessed using the Shapiro-Wilk test. A two-way (load factor × duration) repeated measures analysis of variance (ANOVA) was employed for statistical analysis of each indicator. When testing interaction effects, Greenhouse-Geisser correction was applied if the sphericity assumption was violated. When significant load × duration interactions were observed, simple effects analysis was conducted for each factor using Bonferroni *post hoc* tests; when interactions were non-significant, only main effects of the two factors were analyzed. Effect sizes were calculated using partial eta-squared (η_p_^2^) to evaluate the magnitude of differences: <0.06 indicating small effect, 0.07–0.14 medium effect, and >0.14 large effect (Cohen, 1988).

## 3 Results

### 3.1 Effects of different SIT combinations on wingate test performance under acute testing conditions

**FIGURE 1:**
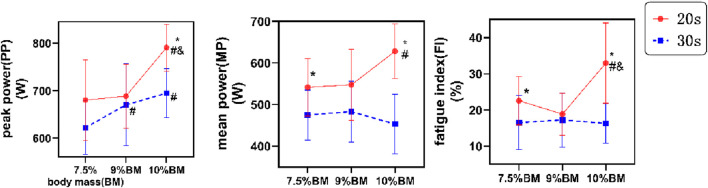
Test results of Wingate with different combinations of SIT. Note: * indicates significant difference compared with 30s, # indicates significant difference compared with 7.5% BM, and and indicates significant difference compared with 9% BM. BM, body mass; FI, fatigue index; MP, mean power; PP, peak power. The same applies below.

The two-way ANOVA results presented in Figure 1 demonstrate that PP, MP, and FI all reached their maximum values under the 20s/10%BM parameter combination. As shown in Table 2, duration exerted a significant main effect on peak power (F (1,22) = 7.242, P = 0.013, η_p_^2^ = 0.248), and highly significant main effects on both mean power (F (1,22) = 17.070, P < 0.001, η_p_^2^ = 0.437) and fatigue index (F (1,22) = 15.186, P < 0.001, η_p_^2^ = 0.408). Load factor showed a significant main effect on mean power (F (2,21) = 5.806, P = 0.010, η_p_^2^ = 0.356), and highly significant main effects on both peak power (F (2,44) = 18.984, P < 0.001, η_p_^2^ = 0.463) and fatigue index (F (2,44) = 6.100, P = 0.005, η_p_^2^ = 0.217). The duration × load interaction effects were statistically significant for all anaerobic power indicators (PP, η_p_^2^ = 0.128, MP, η_p_^2^ = 0.621, FI, η_p_^2^ = 0.259).

### 3.2 Effects of different SIT combinations on wingate test performance under acute testing conditions

**FIGURE 2:**
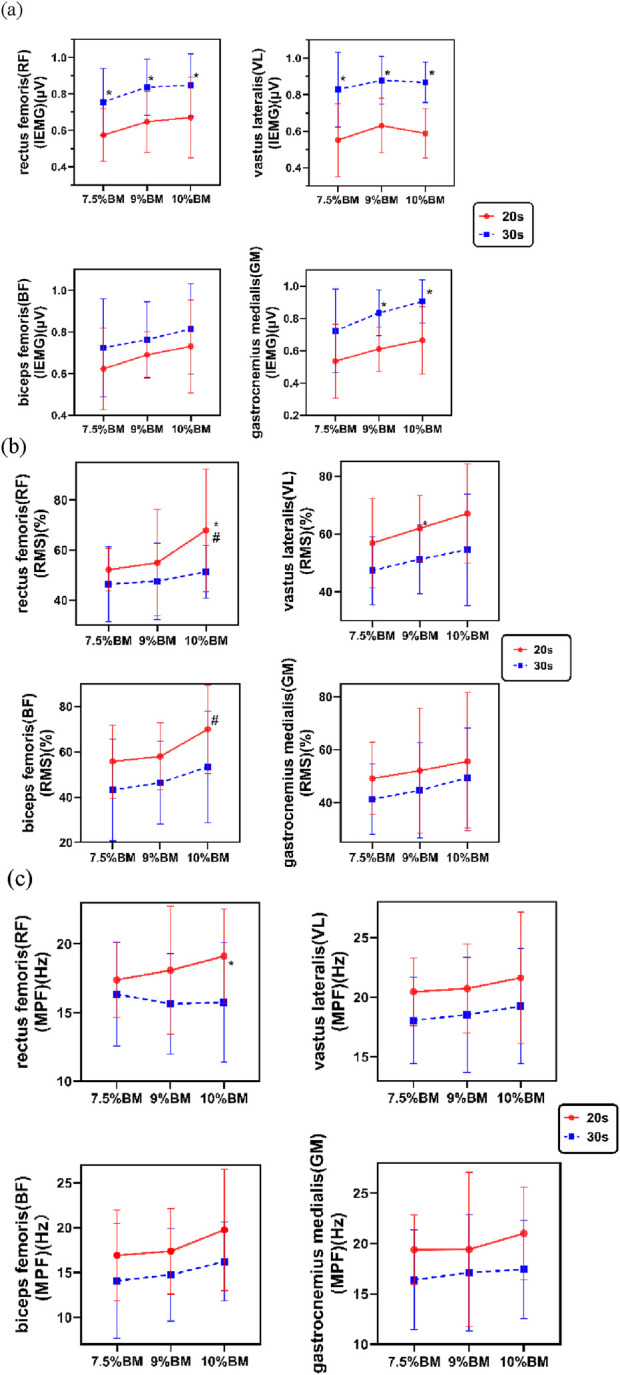
This is a figure. **(a)** Test results of IEMG with different combinations of SIT; **(b)** Test results of RMS% with different combinations of SIT; **(c)** Test results of MPF with different combinations of SIT.

The two-way ANOVA results presented in Figure 2 demonstrate that 30s trials consistently produced greater iEMG values compared to 20s trials, with both durations reaching peak electrical activity under the 10%BM condition. As shown in Table 3, duration exerted highly significant main effects on iEMG for the RF, VL, and GM (P < 0.01), while showing a significant main effect for the BF (P < 0.05). Load factor showed no significant main effects on lower limb muscle iEMG (P > 0.05). Furthermore, no significant duration × load interaction effects were observed for any lower limb muscles’ iEMG (P > 0.05).

**TABLE 3:** A Summary of the results of two-factor ANOVA on muscle IEMG for Different combinations of SIT (n = 12).

	RF		BF		VL		GM	
	F	P	F	P	F	P	F	P
Duration	12.912	0.002	2.767	0.110	60.598	<0.001	20.023	<0.001
Load	2.688	0.79	1.683	0.197	0.943	0.397	3.081	0.067
Interaction effect	0.011	0.989	0.033	0.947	0.072	0.930	0.084	0.920

Note: RF, rectus femoris; VL, vastus lateralis; BF, biceps femoris; GM, gastrocnemius medialis; F, F-statistic; P, P-value. The same applies below.

The two-way ANOVA results presented in Figure 2 demonstrate that 20s trials consistently produced higher RMS% values compared to 30s trials, with both durations reaching peak muscle activation levels under the 10%BM condition. As shown in Table 4, duration showed significant main effects on RMS% for the RF, BF, and VL (P < 0.05), while its effect on GM RMS% was non-significant (P > 0.05). Load factor demonstrated significant main effects on RMS% for the RF, BF, and VL muscles (P < 0.05), but no significant effect on GM RMS% (P > 0.05). No significant duration × load interaction effects were observed for RMS% in any of the tested muscles (P > 0.05).

**TABLE 4:** A Summary of the Results of two-factor ANOVA on muscle RMS% for Different Combinations of SIT (n = 12).

	RF		BF		VL		GM	
	F	P	F	P	F	P	F	P
Duration	5.064	0.035	6.048	0.022	5.483	0.029	1.379	0.253
Load	4.236	0.028	3.294	0.046	3.462	0.040	1.350	0.270
Interaction effect	1.125	0.344	0.150	0.861	0.102	0.904	0.019	0.982

The two-way ANOVA results presented in Figure 2 demonstrate that the 20s trials consistently yielded higher MPF values compared to the 30s trials, with both durations generally reaching their maximum values under the 10%BM condition. As shown in Table 5, duration exhibited significant main effects on MPF for all lower limb muscles (P < 0.05), whereas load factor showed no significant main effects on MPF (P > 0.05). Furthermore, no significant duration × load interaction effects were observed for MPF in any of the lower limb muscles (P > 0.05).

**TABLE 5:** A Summary of the Results of two-factor ANOVA on Muscle MPF for Different Combinations of SIT (n = 12).

	RF		BF		VL		GM	
	F	P	F	P	F	P	F	P
Duration	4.499	0.045	4.621	0.043	4.335	0.049	4.621	0.043
Load	0.224	0.801	1.495	0.235	0.527	0.594	0.428	0.654
Interaction effect	0.718	0.493	0.050	0.952	0.004	0.996	0.091	0.913

### 3.3 Effects of different SIT combinations on blood lactate and RPE test results under acute testing conditions

**FIGURE 3:**
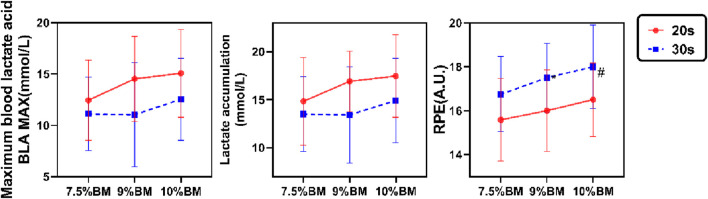
Test results of lactate accumulation, Maximal blood lactate and RPE with different combinations of SIT.

The two-way ANOVA results presented in Figure 3 demonstrate that both maximal blood lactate and accumulated lactate values were significantly higher in 20s trials compared to 30s trials, with peak values for both durations observed under the 10%BM condition. Correspondingly, RPE values also reached their maximum under this condition, though 30s trials yielded higher RPE than 20s trials. As shown in Table 6, duration exhibited significant main effects on maximal lactate, accumulated lactate and RPE (all P < 0.05). Load factor showed no significant main effects on either lactate parameter (both P > 0.05) but significantly influenced RPE (P < 0.05). No significant duration × load interaction effects were observed for any of these parameters (all P > 0.05).

**TABLE 6:** A summary of the results of two-factor ANOVA analysis of blood lactate and RPE for different combinations of SIT (n = 12).

	Maximal blood lactate acid (BLA max)		Lactate accumulation		RPE	
	F	P	F	P	F	P
Duration	5.469	0.029	5.983	0.023	6.108	0.022
Load	1.425	0.251	1.425	0.251	3.781	0.031
Interaction effect	0.405	0.669	0.405	0.669	0.119	0.888

**Note:** BLA, max, Maximal blood lactate acid.

## 4 Discussion

This study compared the acute physiological characteristics of different SIT protocols to investigate the effects of acute experimental variables and their interactions on anaerobic capacity in 200-m sprinters, aiming to identify the optimal SIT combination for maximizing anaerobic performance benefits and provide theoretical and experimental foundations for the appropriate application of sprint interval training in these athletes. The results demonstrate that an SIT protocol consisting of 20-s sprints at 10% BM load with 4-min inter-set recovery intervals (4 sets per session) elicits superior anaerobic capacity development.

### 4.1 Analysis of different SIT combinations on wingate test performance under acute testing conditions

The results of this study demonstrate that during the Wingate test, four maximal 20-s sprints at 10% BM load elicited peak values for both PP and MP, while simultaneously producing the highest FI, indicating maximal fatigue. These findings suggest that this protocol enables athletes to achieve optimal explosive power and mean power output, albeit with concomitantly greater muscular fatigue. Furthermore, significant duration × load interaction effects were observed across all Wingate test parameters, revealing that the influence of different training loads on performance metrics varies significantly over time.

Regarding the impact of sprint duration on training effects, our findings align with research by Yamagishi et al. (2024) and Hazell et al. (2010), consistently demonstrating that shorter-duration sprints (20s) in SIT protocols yield superior peak and mean power outputs compared to longer sprints (30s). The 20s SIT group exhibited the most pronounced anaerobic capacity enhancement among all test conditions, a finding with significant practical implications - optimizing sprint duration can substantially improve training efficiency by achieving higher anaerobic performance gains within shorter timeframes (Balsom et al., 1992). In our study, all 20s sprint conditions produced significantly greater PP and MP than 30s protocols, suggesting that reduced duration SIT-based Wingate testing may not compromise training efficacy but rather enhance athletic performance. From an energy metabolism perspective, the glycolytic system predominates during 20-s sprints, with its contribution progressively increasing with duration. Shorter sprints conserve glycogen reserves while facilitating phosphocreatine (PCr) resynthesis and enhancing glycolytic enzyme activity, thereby sustaining peak performance and providing metabolic substrates for subsequent sprints explaining why glycolysis serves as the primary driver in SIT (Hazell et al., 2010). Regarding muscle fiber recruitment, brief high-intensity efforts preferentially activate fast-twitch fibers through rapid, extensive recruitment. Although these fibers exhibit higher FI due to their rapid fatigability and recovery characteristics, this pattern better meets the explosive power demands of sprint events. Conversely, prolonged high-intensity efforts increasingly engage aerobic metabolism (Bompa, 2015), leading to declining power output and inferior anaerobic performance explaining the poorer outcomes in our 30s protocols. Furthermore, sustained efforts progressively recruit slow-twitch fibers, which may reduce overall FI but prove less effective for developing sprint-specific anaerobic capacity.

Regarding the impact of load intensity on training effects, the present findings demonstrate that under 20-s sprint conditions, the 10%BM SIT protocol significantly outperformed other loading schemes in both peak and mean power outputs. From an energy metabolism perspective, sustained ATP supply during exercise is crucial for skeletal muscle contraction and maintenance of continuous movement (Hargreaves and Spriet, 2020). Athletes in this discipline exhibit superior ATP production capacity, with the 10%BM load optimally enhancing ATP-CP system efficiency, enabling simultaneous high-intensity output and maximal utilization of muscular and blood buffering capacities (Metcalfe et al., 2018). Neuromuscular regulation analysis reveals that increased loading preferentially recruits fast-twitch fibers, whose inherent contraction velocity and force generation characteristics potentiate explosive power performance. At the neural drive level, sprinting enhances muscle contraction efficiency through synchronized firing and increased discharge frequency, thereby boosting instantaneous power output. Conversely, under 30-s sprints, the 10%BM condition paradoxically yielded the lowest power outputs. This phenomenon likely reflects progressive slow-twitch fiber recruitment whose metabolic properties - while supporting endurance - reduce overall power production, consequently manifesting as decreased MP. The FI analysis showed a slight decline at 20s/9%BM, potentially attributable to compensatory fiber recruitment mechanisms under suboptimal loading, where alternating muscle fiber activation maintains pedaling force by replacing fatigued fibers (Krustrup et al., 2004). Higher loads accelerate PCr depletion and lactate accumulation, exacerbating fatigue. However, prolonged 30-s sprints exhibited reduced FI values, indicating slower fatigue accumulation due to increased aerobic contribution while promoting metabolic stability, this adaptation proves suboptimal for maximal anaerobic capacity development.

### 4.2 Analysis of different SIT combinations on electromyographic activity under acute testing conditions

In EMG testing, iEMG represents the time-integrated absolute amplitude of EMG signals, quantifying the total electrical activity of muscles. The EMG results demonstrated significantly higher iEMG values across all measured muscles during 30-s sprints compared to 20-s sprints. This phenomenon relates to phase-dependent variations in central nervous system (CNS) stimulation and differential muscle fiber recruitment kinetics (Zhou and Yang, 1996). The CNS compensates for declining muscle force by progressively increasing discharge rates of motor cortex neurons, which enhances spinal α-motoneuron excitability, thereby augmenting both motor unit recruitment and firing frequency collectively elevating iEMG. Existing research confirms a robust linear relationship between changes in muscle contraction force/power output and sEMG amplitude across various loading conditions (Wang, 2000). Our findings in RF, BF, and GM muscle activities corroborate this conclusion. From a biomechanical perspective, as primary power generating muscles during cycling, the RF and BF require enhanced electrical activity to increase pedaling force, while the GM crucial for ankle plantarflexion exhibits load-dependent EMG amplification. However, studies indicate that iEMG increases do not maintain perfect linearity with loading but demonstrate abrupt surges within specific load ranges, reflecting rapid mass recruitment of fast twitch fibers at intensities approximating the anaerobic threshold (Fatela et al., 2016). This pattern emerged in our study, where VL showed marked iEMG increases at 9%BM but plateaued or slightly decreased at 10%BM, suggesting near maximal muscle activation for knee extension at lower loads with subsequent contribution saturation upon further loading.

Electromyographic analysis revealed that RMS, as a key indicator of muscle activation level, effectively reflects neuromuscular adaptive changes under different exercise conditions. During movement, muscle activity levels continuously adjust in response to functional demands and exercise duration (Parsaei et al., 2010). The BF demonstrated significant time dependent activation characteristics, exhibiting higher RMS% values compared to other muscles, indicating greater relative contribution and recruitment of motor units. In contrast, the GM showed lower activation levels due to its smaller functional contribution, displaying relatively weaker sensitivity to SIT. Regarding exercise duration effects, brief sprints triggered synchronous recruitment of numerous fast twitch fibers with high discharge frequencies and amplitudes, resulting in markedly elevated RMS values. During 30-s SIT, PCr depletion coupled with lactate and H^+^ accumulation from glycolysis impaired contractile capacity, ultimately reducing overall muscle activation. Load intensity differentially affected lower limb motor unit activation, likely mediated through load-dependent modulation of muscular coordination and neural drive (Loris and Wang, 2017). Our observations showed progressive RMS% increases with loading, peaking at 10%BM. Specifically, the RF and BF exhibited significantly greater activation at 10%BM versus 9%BM. This reflects RF’s lower activation threshold under femoral nerve innervation achieving full fiber recruitment at moderate loads with increased firing rates, and BF’s greater fast-twitch fiber composition enabling enhanced unit recruitment at higher loads (Person and Kudina, 1972) (Reece et al., 2021). Notably, the VL and GM demonstrated comparatively lower activation, potentially attributable to their distinct functional roles and neurophysiological properties during cycling motions.

MPF, a standard sEMG spectral analysis parameter, reflects neurophysiological characteristics of exercise-induced muscle fatigue (Jurell, 1998). Research indicates that lower limb muscles exhibit distinct fatigue patterns during different exercise modalities (Xiang, 2010), primarily attributable to variations in fiber-type composition and metabolic properties. During short-duration, high-intensity SIT, the nervous system preferentially recruits fast-twitch fibers with rapid conduction velocities and high discharge frequencies to meet instantaneous power demands, consequently increasing MPF. As fast-twitch fibers fatigue, the body compensates by recruiting slower-conducting but fatigue-resistant slow-twitch fibers to sustain high-load exercise (Henneman et al., 1965), (Dankel et al., 2017). Notably, 200-m sprinters possess inherently greater fast-twitch fiber proportions to meet event-specific demands. This morphological advantage enables greater initial recruitment of fast-twitch motor units, generating higher baseline MPF values. Studies demonstrate that larger MPF decrements correlate with reduced recruitment of high-threshold motor units (Dankel et al., 2017). Our findings of systematically lower MPF during 30-s versus 20-s sprints suggest impaired maintenance of high-threshold unit recruitment during prolonged efforts (Wang et al., 2015), neurophysiological evidence supporting greater neuromuscular fatigue during 30-s sprints.

Furthermore, investigation of duration - load interactions on muscular electrical activity revealed no significant synergistic effects between these factors. Recovery intervals allowing temporal and loading factors to operate relatively independently. However, subsequent main effect analysis demonstrated that sprint duration significantly influenced electromyographic characteristics, specifically modulating total fiber discharge, activation levels, and fatigue development. In contrast, load intensity variations exhibited comparatively limited regulatory effects on these EMG parameters. This suggests that, given adequate recovery, the neuromuscular system displays markedly greater sensitivity to sprint duration than to load intensity variations.

### 4.3 Analysis of blood lactate and RPE responses to different SIT combinations under acute testing conditions

From a metabolic perspective, the 20-s SIT protocol significantly enhanced lactate production capacity, as evidenced by marked increases in both maximal and accumulated blood lactate levels, consistent with existing research (Yin et al., 2025) (Wood et al., 2016). Specifically, four 20-s sprints strongly stimulated the glycolytic system, resulting in substantially elevated lactate accumulation. The heightened glycolytic flux in fast twitch fibers, combined with fatigue induced H^+^ accumulation that inhibits key enzymatic activity, creates a metabolic vicious cycle exacerbating physiological stress (Hargreaves and Spriet, 2020). Metabolically, SIT exhibits characteristic mixed aerobic anaerobic properties: initial dominance of anaerobic pathways gradually shifts toward aerobic contribution. However, the 4-min recovery intervals allow near complete phosphagen system replenishment, maintaining subsequent sprint performance while permitting progressive lactate accumulation (Yin, 2023) (Sun et al., 2018). Notably, load intensity demonstrated relatively limited effects on lactate responses, likely because brief sprints primarily engage the phosphagen system initially, while glycolytic activation exhibits temporal delay, buffering immediate load-dependent lactate variations.

The RPE serves as a subjective assessment of an individual’s fatigue or effort level during exercise, demonstrating high consistency with physiological load indicators. This study clearly illustrates a linear relationship between temporal factors and RPE, where prolonged duration and increased load proportionally elevate perceived fatigue. Relevant studies have demonstrated that higher mechanical loads may temporarily suppress cortical processing of fatigue perception through enhanced activation of central motor commands, resulting in the “power-perception dissociation” phenomenon (Amann, 2011). Notably, 20-s sprints elicited lower fatigue perception. From an exercise psychology perspective, shorter duration sprint training induces more positive affective responses, including greater training enjoyment and participation willingness. Conventional 30-s SIT protocols are consistently perceived as more aversive (Hardcastle et al., 2014) (Biddle and Batterham, 2015), while abbreviated sprints generate more favorable psychological reactions (Martinez et al., 2015; McKie et al., 2018; Metcalfe et al., 2022). This aligns with Townsend et al.'s findings (Townsend et al., 2017) demonstrating enhanced positive psychological effects with repeated short sprints a potentially more pleasurable alternative to prolonged endurance training (Bartlett et al., 2011) (Kong et al., 2016). Mechanistically, intensity dependent physiological overload elevates metabolic stress, directly amplifying subjective fatigue perception (Hu et al., 2020). Research indicates that under specific conditions, certain fatigue sensations may dominate subjective perception while others become attenuated. In cycling exercise, fatigue perception is primarily regulated by local factors (muscular fatigue). This mechanism can explain the findings of the present study: during 30-s sprint intervals, decreased muscle MPF values were accompanied by elevated RPE scores (Pandolf et al., 1975). Consequently, both temporal and loading parameters significantly influence athletes’ subjective experience, underscoring the necessity of monitoring perceptual responses in training prescription.

### 4.4 Limitations

Given the documented sex differences in hormonal profiles, substrate utilization, and fatigue resistance between male and female athletes, these findings may not be generalizable to female competitors. Consequently, future studies should specifically investigate anaerobic capacity adaptations in female athletes using comparable SIT protocols. Given that this would allow for a more comprehensive analysis of physiological responses, it is recommended to incorporate heart rate monitoring. Additionally, while the study examined acute responses, the long-term adaptive effects of the proposed SIT protocol remain to be validated through longitudinal training studies.

## 5 Conclusion

This study confirms that an acute sprint interval protocol consisting of 4 maximal 20-s sprints at 10% BM load with 4-min recovery intervals provides multidimensional stimulation of anaerobic capacity in 200-m sprinters. The SIT protocol under these conditions significantly activates both the ATP-PCr system and glycolytic pathways, effectively enhancing not only peak power output but also speed endurance performance. EMG analysis reveals its specific capacity to augment neuromuscular activation in the biceps femoris and rectus femoris, while simultaneously inducing greater lactate accumulation. This dual neuro-metabolic mechanism establishes the protocol as an effective training strategy for optimizing anaerobic performance in 200-m specialists.

## Data Availability

The original contributions presented in the study are included in the article/supplementary material, further inquiries can be directed to the corresponding author.
